# Pharmacogenetic Testing of Cytochrome P450 Drug Metabolizing Enzymes in a Case Series of Patients with Prader-Willi Syndrome

**DOI:** 10.3390/genes12020152

**Published:** 2021-01-24

**Authors:** Janice Forster, Jessica Duis, Merlin G. Butler

**Affiliations:** 1 Pittsburgh Partnership, PWS, Pittsburgh, PA 15218, USA; 2 Section of Genetic and Inherited Metabolic Disease, Department of Pediatrics, Children’s Hospital Colorado, Aurora, CO 80045, USA; jessica.duis@childrenscolorado.org; 3 Division of Research and Genetics, Departments of Psychiatry & Behavioral Sciences and Pediatrics, University of Kansas Medical Center, Kansas City, KS 66160, USA; mbutler4@kumc.edu

**Keywords:** pharmacogenetic testing, cytochrome P450 enzymes, Prader-Willi syndrome, drug interactions, medication management

## Abstract

Prader-Willi syndrome (PWS) is associated with co-morbid psychiatric symptoms (disruptive behavior, anxiety, mood disorders, and psychosis) often requiring psychotropic medications. In this clinical case series of 35 patients with PWS, pharmacogenetic testing was obtained to determine allele frequencies predicting variations in activity of cytochrome (CYP) P450 drug metabolizing enzymes 2D6, 2B6, 2C19, 2C9, 3A4, and 1A2. Results were deidentified, collated, and analyzed by PWS genetic subtype: 14 deletion (DEL), 16 maternal uniparental disomy (UPD) and 5 DNA-methylation positive unspecified molecular subtype (PWS Unspec). Literature review informed comparative population frequencies of CYP polymorphisms, phenotypes, and substrate specificity. Among the total PWS cohort, extensive metabolizer (EM) activity prevailed across all cytochromes except CYP1A2, which showed greater ultra-rapid metabolizer (UM) status (*p* < 0.05), especially among UPD. Among PWS genetic subtypes, there were statistically significant differences in metabolizing status for cytochromes 2D6, 2C19, 2C9, 3A4 and 1A2 acting on substrates such as fluoxetine, risperidone, sertraline, modafinil, aripiprazole, citalopram, and escitalopram. Gonadal steroid therapy may further impact metabolism of 2C19, 2C9, 3A4 and 1A2 substrates. The status of growth hormone treatment may affect CYP3A4 activity with gender specificity. Pharmacogenetic testing together with PWS genetic subtyping may inform psychotropic medication dosing parameters and risk for adverse events.

## 1. Introduction

### 1.1. Prader-Willi Syndrome (PWS)

PWS is a rare, complex genetic disorder recognized as the most common form of syndromic obesity. PWS is reported to affect between 350,000 and 400,000 individuals worldwide with an estimated prevalence of one in 10,000 to 38,000 individuals [1]. PWS results from errors in genomic imprinting with loss of expression of paternal genes in the chromosome 15q11–q13 region generally from a paternal deletion (DEL, 60% of cases) followed by maternal uniparental disomy 15 (UPD, 35% of cases) in which both chromosome 15 s are inherited from the mother. Imprinting center defects (IC) and chromosome 15 translocations or inversions are seen in the remaining patients [2]. This multi-system disorder is characterized by severe infantile hypotonia with a poor suck, weak cry, failure to thrive and feeding difficulties. Hypogonadism/hypogenitalism is noted at birth. Hypothalamic dysfunction causes growth and other hormone deficiencies, as well as dysregulation of body temperature, sleep and wakefulness, hunger, thirst, and stress response. Currently, growth hormone, usually started in the first year of life, is the only medication indicated for PWS to manage short stature, small hands and feet, global developmental delay and abnormalities of body composition consisting of excessive fat mass to lean body weight [3]. In early childhood there is an increased interest in food, and calories must be managed to avoid excess weight gain. Six nutritional phases have been described [4]. Excessive food intake leads to obesity, unless dietary management, environmental controls and mandatory exercise are implemented. Psychological food security helps to manage behavior difficulties presenting around food [5]. Temper tantrums emerge in early childhood and persist into adulthood. They are followed by the appearance of other behavior problems in middle childhood. These include manifestations of cognitive rigidity (e.g., repetitive asking, insistence on sameness, selective attention, difficulty with transitions), and anxiety (e.g., excessive and repetitive actions, emotional lability, and stress sensitivity) [6]. These characteristics define the behavioral phenotype of PWS and are often a focus of treatment from mental health professionals. Behavior management, psychological therapies and psychotropic medications are often prescribed. Symptoms of co-morbid psychiatric conditions (e.g., mood disorder and psychosis) often emerge in adolescence and require medication management [6]. There are some phenotypic differences that correlate with PWS genetic subtypes. Individuals with the deletion subtype are generally more affected with compulsions and self-injury and perform more poorly on cognitive and behavior instruments [7,8]. Those with UPD have higher verbal IQs than those with deletion, but they may be more prone to symptoms of autism spectrum disorder in early childhood and affective psychosis with onset in adolescence and young adult years [9,10]. There is an increased incidence of psychosis among both subtypes [10,11].

The NIH Rare Disease PWS Consortium Registry tracked 355 patients during clinic visits over ten years and recorded age of onset of use of psychotropic medication and pattern of sustained utilization [12]. A total of 265 patients were receiving 483 psychotropic medications. From these data, it appears that multiple medications were used for management of the complex symptoms and co-morbid conditions associated with PWS [13]. Selective serotonin reuptake inhibitors (SSRIs) were used in nearly 50% of the 5–12 year age group and in 70% of the 12–21 year age cohort. Atypical neuroleptics were the second most frequent class of medications used (34%), often in combination with SSRIs [13]. Polypharmacy, the use of more than one medication of a single class or multiple medications from different classes, increases the risk for drug-drug interactions and adverse effects, and this tendency is exacerbated at younger ages. Careful medication selection and informed medication management is required [14].

### 1.2. Pharmacogenetics of the Cytochrome P450 System

Pharmacogenetics examines the influence of DNA structural variations on genes coding for enzymes responsible for drug metabolism determining efficacy and tolerability. These protein-coding genes are diverse across the genome. The cytochrome P450 enzyme system (CYP) is a heme-based superfamily of proteins responsible for the oxidative phosphorylation of toxins and medications and the synthesis of lipids, steroids (hormones) and some vitamins [14]. This enzyme system is present in most body tissues including the liver and brain [15,16]. It is primarily positioned within the inner mitochondrial membrane or endoplasmic reticulum of the cell. Up to 80% of all drugs are metabolized in the liver by these six different cytochrome P450 enzymes: CYP1A2, CYP2B6, CYPC19, CYP2D6, CYP3A4 and CYP3A5 [17]. CYPD26 by itself may account for the breakdown of up to 25% of all medications [14]. Gene variants have clinically relevant impact on drug metabolism, drug efficacy, side effects, and drug-drug interaction in the clinical setting; they are also associated with susceptibility to cancer and disease [18]. Drugs undergoing metabolism often involve more than one cytochrome enzyme. This is graphically represented for most drugs [https://www.pharmgkb.org/pathways]. In addition, drugs may require first pass metabolism to generate the therapeutic agent for treatment effect. Also, cytochrome P450 genes and their encoded enzymes may be altered by the environment through inhibitors and inducers [14]. The chromosome location for genes encoding cytochrome P450 enzymes that are discussed in this article, their common polymorphisms, and phenotypic activity can be found in Table A1. The Variation (PharmVar) Consortium: Incorporation of the Human Cytochrome P450 (CYP) Allele Nomenclature Database [https://www.pharmgkb.org] is a helpful reference to understand and decode gene notation, population frequency and phenotypic expression.

Compared to all medications, psychotropic drugs are more selectively metabolized by cytochromes 2D6, 2B6, 3A4, 1A2, 2C19 and 2C9 [19]. These genes have many polymorphisms that produce enzymes of variable activity. Some alleles have enhanced activity while others are reduced or inactive. The cytochrome P450 phenotype is defined by the number and combination of alleles inherited from the parents. Gene function is described by four phenotypic categories: ultra-rapid metabolizer (UM); extensive metabolizer (EM); intermediate metabolizer (IM); and poor metabolizer (PM) for each cytochrome P450 gene. The typical or normal rate of activity is the extensive metabolizing phenotype.

Also, gene polymorphisms vary according to race, ethnicity, and geographical ancestry. A comprehensive list of gene polymorphisms and their phenotypic activity are reported at [https://www.pharmgkb.org]. Similar data, together with allelic frequencies and their racial and ethno-geographic frequency, can be found in the review article by Zhou et al. [20].

More than ten years ago at Vanderbilt University, a survey was completed by the parents or caregivers of 86 persons with PWS to ascertain the respondent’s satisfaction with the use of SSRIs and/or atypical neuroleptics to manage behavioral symptoms related to PWS [21]. SSRIs were used in 33%, atypical neuroleptics were used in 11%, and a combination of SSRIs plus atypical antipsychotics was used in 17% of the study population. PWS genetic subtype was not specified. In this pilot study, research-based probes were used to identify phenotypes of CYP2D6 and CYP2C19; allelic frequencies were not reported. Although the EM phenotype predominated for cytochromes 2D6 and 2C19, 37% were IMs of CYP2D6, 2.5% were PMs of CYP2D6, and 3.2% were PMs of CYP2C19. The results of the Vanderbilt study indicated that in more than one-third of persons with PWS, the IM or PM status was noted for CYP2D6. It can be inferred that serum levels of antidepressant or antipsychotic medication metabolized by CYP2D6 would be higher than expected at typical doses, leading to clinical response at lower doses or adverse effects at typical doses. This finding is corroborated by clinical experience with SSRI medications in PWS, as described in the mood and behavioral activation case series reported by Durette et al. [22].

The aim of this clinical report was to examine and summarize pharmacogenetic results from a cohort of patients with PWS referred for evaluation and treatment of psychiatric and behavioral problems. Our approach was to identify differences in CYP genotypes and phenotypes in the referred cohort as a whole, correlate these findings with phenotypic frequencies among the PWS genetic subtypes, and compare these results to data from a normative population. These findings may inform our understanding of why many of our patients have a therapeutic response at low doses of SSRIs and adverse events at typical doses of psychotropic medications as reported by Gourash et al. [23]. This knowledge will be used in our daily clinical practice to guide selection and dose of psychotropic medication, to anticipate potential drug interactions, and to foresee vulnerability to adverse effects while treating the psychiatric and behavioral problems occurring in our patients with this rare genetic syndrome.

## 2. Methods

Thirty-five patients with PWS (14 DEL; 16 UPD; 5 Unspec-methylation positive, molecular subtype unspecified) were seen for evaluation and treatment at one of three regional centers by the physician authors who have extensive experience using psychotropic medications to manage psychiatric or behavior problems in patients with PWS. The clinical use of pharmacogenetic testing was discussed with and approved by the patient and/or guardian, and testing was ordered to determine cytochrome P450 function to guide the selection of medication and the dosage required for treatment. The authors collected buccal cells in the clinical setting and sent them to one of three CLIA approved and accredited, commercial laboratories: Genesight (GS) in Mason, Ohio (*n =* 29); Genelex (GL) in Seattle, Washington (*n =* 5); and Genomind (GM) in King of Prussia, Pennsylvania (*n =* 1). These laboratories undertake quality control testing required for accreditation to assure and maintain accuracy of laboratory testing results. DNA was extracted at the laboratories and analyzed for polymorphisms of *CYP2D6*, *CYP2B6*, *CYP2C19*, *CYP2C9*, *CYP3A4* and *CYP1A2* as well as other genes not included in this case report. Results were received by the authors and protected health information was removed (name, age, gender, race, ethnicity) prior to data pooling and sorting by genetic subtype of PWS. Psychiatric diagnosis, psychotropic medication history, and family psychiatric and treatment history were not available, although all the patients met medical necessity criteria indicating a failure of previous medications, either due to inefficacy or adverse effects, and/or the presence of psychiatric co-morbidities that would require treatment with multiple psychotropic medications.

When comparing the testing results from the three commercial laboratories, there were subtle differences noted. In some cases, different nomenclature was used for the same results, i.e., CYP1A2 *1 and *1A are both names for the wildtype gene, and CYP1A2*1F is the hyper-inducible-163 A/A polymorphism. Also, we found that the interpretation of phenotype from genotype may differ across the commercial laboratories, as each of them uses a combinatorial phenotype that is determined by a proprietary algorithm. For example, GL identifies any carrier of CYP1A2*1F as HI (hyper-inducible), while GS identifies *1F heterozygotes as UM (ultrarapid metabolizers) and GM identifies them as EM (extensive metabolizers). Although we used the phenotypic nomenclature reported by the commercial companies designating metabolizing status as poor, intermediate, extensive and ultrarapid, we also analyzed the frequencies of alleles and diplotypes and compared them to published norms.

For *CYP2C19*, the pharmacogenomic companies reported *CYP2C19* phenotype as ultrarapid, extensive, intermediate, and poor metabolizing. However, the authors were aware that the current phenotypic nomenclature for *CYP2C19* has been changed to ultrarapid, rapid, normal, intermediate and poor metabolizing, as described at https://www.pharmgkb.org/page/cyp2c19RefMaterials, to more accurately describe the function of the CYP2C19*17 rapid metabolizing allele. To calculate the phenotypic frequencies for *CYP2C19* from the normative data, we combined the frequencies for rapid and normal metabolizing phenotype and used this value for the normative extensive metabolizers, as discussed and implemented in Martis et al. [24]. Pharmacokinetic testing has indicated that there is minimal variance between the normal and rapid metabolizing phenotypes [25].

In this clinical case report, the frequency of phenotypes assigned by the commercial providers for each cytochrome P450 gene is displayed as a histogram for each PWS genetic subtype (DEL, UPD and PWS Unspec) and compared to frequencies found in the normative Caucasian population. Then, for each genetic subtype of PWS, the frequency of phenotypes for each CYP gene was calculated, compared to published normative data, and analyzed for significance using the chi-square test. *p* values of <0.05 were statistically significant. Next, for each cytochrome P450 polymorphism, the frequency of occurrence of the alleles and diplotypes was calculated for each genetic subtype of PWS and displayed graphically for comparison with normative population data.

For this case series normative data from the Caucasian population was referenced because the NIH PWS Registry found minimal racial/ethnic diversity (93% Caucasian) among 355 enrollees from regional clinics across the USA. The phenotypic frequency in the Caucasian population was obtained from https://www.pharmgkb.org for cytochromes *2D6*, *2B6*, *2C19*, and *2C9*, and Zhou et al. was used for *3A4* [20]. The values reported in the literature for the frequencies of CYP phenotypes for each cytochrome show some variability across studies even within designated ethnic categories. Normative data for the phenotypic frequency of *1A2* among Caucasians was not available, although the increased prevalence of the poor metabolizer phenotype among the Asian population is well documented in the literature. We elected to use the results of studies measuring urinary caffeine metabolites to report the normative metabolic phenotype of CYP1A2, a method that has been used for over 20 years and more recently correlated with genotype [26,27,28].

The patients in this clinical case series sought care at one of three specialty programs across the USA. They were evaluated by physician experts with over 80 years of collective clinical experience in treating patients with this rare disorder. These patients received pharmacogenetic testing as part of their medical evaluation because it was deemed as medically necessary. The clinical care described in this article was not part of a research project, so this report did not require ethical review or approval. Prior to the collation and analysis of data, all pertinent private or protected health information about each patient was eliminated or deidentified, except for the genetic subtype of PWS and the pharmacogenetic genotypes and phenotypes. The results of the analysis of this group data will not affect the patient’s clinical care, nor does it have the potential to cause the patient any harm. It is the authors hope that this report will inform, improve, and advance the quality of medical and psychiatric care of patients with PWS.

## 3. Results

This case series represents the largest number of patients with known genetic subtype of PWS to receive pharmacogenetic testing with analysis of results that are summarized in Table 1. The frequencies of CYP phenotypes in the PWS cohort are itemized for each genetic subtype and compared to a normative (typical) Caucasian population. The list of substrates affected by these CYP phenotypic differences was derived from the frequency of medication use among 265 patients with PWS enrolled in the NIH PWS Registry [12,13]. The raw data obtained from the pharmacogenetic testing of this cohort of referred patients is displayed in the Appendix A. For each genetic subtype of PWS, the testing laboratory, cytochrome P450 genotype, and cytochrome P450 phenotype are specified in a series of tables: Deletion (Table A2), UPD (Table A3) and PWS Unspecified (Table A4). Table A5 shows the proportion of phenotypes for each cytochrome P450 gene according to the PWS genetic subtype.

### 3.1. Cytochrome P450 Genotypes, Phenotypes and Genetic Subtype of PWS

**Figure 1:**
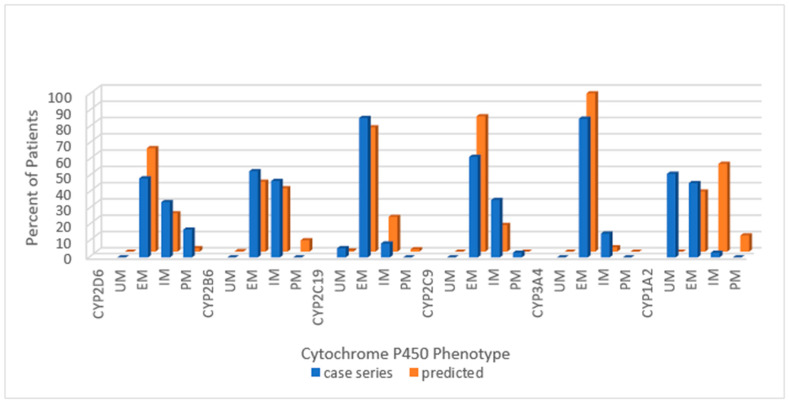
Distribution of Cytochrome P450 phenotypes among the combined PWS cohort (*n =* 35) compared to predicted, normative populations [https://www.pharmgkb.org/page/cyp2d6RefMaterials]; [https://www.pharmgkb.org/page/cyp2b6RefMaterials]; [https://www.pharmgkb.org/page/cyp2c19RefMaterials]; [https://www.pharmgkb.org/page/cyp2c9RefMaterials]; [20,27]. Key: UM—ultra-rapid metabolizer; EM—extensive metabolizer; IM—intermediate metabolizer; PM—poor metabolizer.

The cytochrome P450 phenotypes for the combined group of patients with PWS in our referred cohort is shown in Figure 1. The distribution of frequencies of cytochrome P450 phenotypes is displayed as a percentage of the total cohort (*n =* 35) for ease of comparison with normative data.

The normative data delineating the phenotypic frequencies for CYP1A2 activity (slow, intermediate and rapid) were derived from studies measuring caffeine and its metabolites in urine [27]. Al-Ahmad et al. have described the correlation between metabolic phenotypes and genotypes, e.g., rapid metabolizer phenotype corresponds to *1A/*1A (extensive metabolizer) genotype [28].

**Figure 2:**
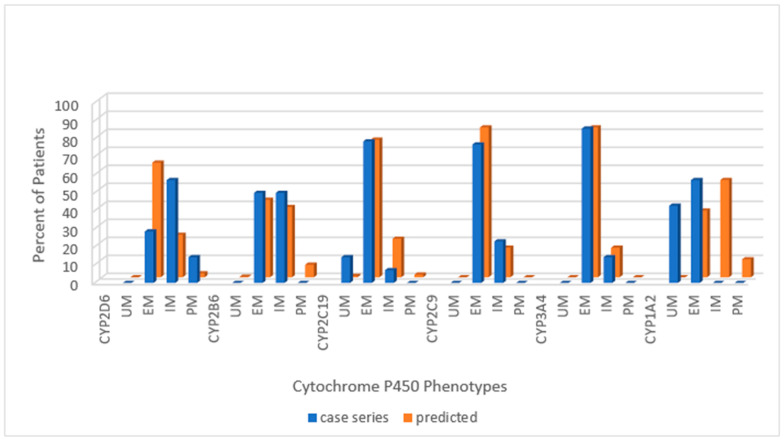
Distribution of Cytochrome P450 phenotypes among PWS DEL (*n =* 14) compared to normative populations [https://www.pharmgkb.org/page/cyp2d6RefMaterials]; [https://www.pharmgkb.org/page/cyp2b6RefMaterials]; [https://www.pharmgkb.org/page/cyp2c19RefMaterials]; [https://www.pharmgkb.org/page/cyp2c9RefMaterials]; [20,27]. Key: UM—ultra-rapid metabolizer; EM—extensive metabolizer; IM—intermediate metabolizer; PM—poor metabolizer.

**Figure 3:**
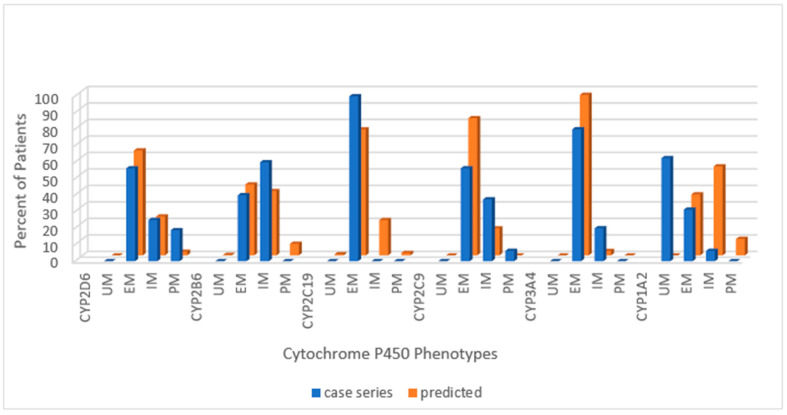
Distribution of Cytochrome P450 phenotypes among the PWS UPD cohort (*n =* 16) compared to normative populations [https://www.pharmgkb.org/page/cyp2d6RefMaterials]; [https://www.pharmgkb.org/page/cyp2b6RefMaterials]; [https://www.pharmgkb.org/page/cyp2c19RefMaterials]; [https://www.pharmgkb.org/page/cyp2c9RefMaterials]; [20,27]. Note: There were 15 results for cytochromes 2B6 and 3A4. Key: UM—ultra-rapid metabolizer; EM—extensive metabolizer; IM—intermediate metabolizer; PM—poor metabolizer.

**Figure 4:**
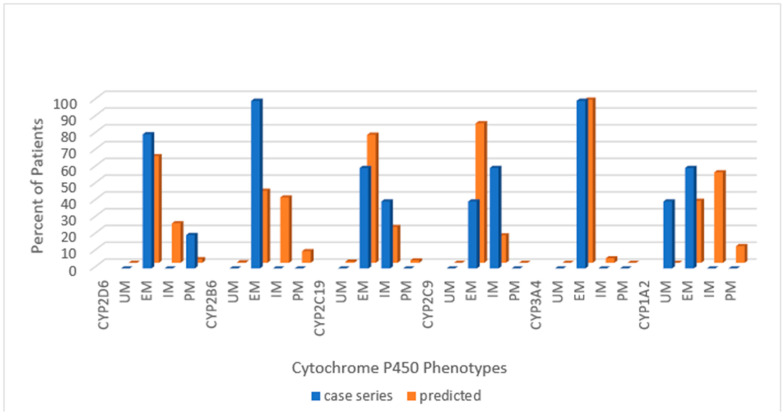
Distribution of Cytochrome P450 phenotypes among PWS Unspecified genetic subtype, (*n =* 5) compared to normative populations [https://www.pharmgkb.org/page/cyp2d6RefMaterials]; [https://www.pharmgkb.org/page/cyp2b6RefMaterials]; [https://www.pharmgkb.org/page/cyp2c19RefMaterials]; [https://www.pharmgkb.org/page/cyp2c9RefMaterials]; [20,27]. Key: UM—ultra-rapid metabolizer; EM—extensive metabolizer; IM—intermediate metabolizer; PM—poor metabolizer.

Across the combined cohort, the extensive metabolizing status prevailed in all but one cytochrome; for CYP1A2, the ultra-rapid phenotype was more common than the extensive metabolizing. Extensive metabolizing phenotype for the predicted normative data exceeded the PWS cohort for CYP2D6 and CYP2C9 but not for CYP2B6 or CYP2C19. Poor metabolizing status for CYP2D6 and intermediate metabolizing status for CYP2C9 were greater in the PWS cohort than predicted in normative populations. The following series of histograms display the cytochrome P450 enzyme phenotypes for each PWS genetic subtype (Figure 2—DEL, Figure 3—UPD, and Figure 4—PWS Unspecified) and compare these to the predicted normative data for CYP2D6, CYP2B6, CYP2C19 and CYP2C9.

When the data sets for PWS genetic subtypes in our referred population were compared to each other, differences were found in the distribution of phenotypes for all cytochromes. In the following sections, the allelic frequencies of the cytochrome P450 gene polymorphisms are displayed for the PWS genetic subtypes. Also, when possible, the distribution of diplotypes is itemized and compared with normative data. The phenotypic action of the most common alleles for each cytochrome P450 gene is itemized in Table A1 (Appendix A).

### 3.2. CYP2D6

For CYP2D6 the data from our case report and the Vanderbilt study is compared to the normative American population referenced at [www.pharmgkb.org/page/cyp2b6RefMaterials]. In our combined cohort of referred patients, 48.6% of had the extensive metabolizing phenotype compared to 63.6% among the normative American population; this was not significantly different by chi-square test (*p* > 0.05). The percentage of CYP2D6 intermediate metabolizers was 34%, and this is similar to the percentage reported in the Vanderbilt survey (37%); both values exceed the 23.6% found in the normative American population, but the chi-square value was not significant (*p* > 0.05). There were 17.1% poor metabolizers in the current cohort compared to 2.5% in the Vanderbilt survey and 2.2% in the normative American population, and the chi-square value was significant (*p* < 0.05). When comparing the current data to the Vanderbilt survey, it should be noted that the current cohort was derived from a clinically referred sample, where medical necessity dictated testing. There may have been more treatment failures or adverse events in the current cohort. Data from our referred cohort indicates that over half of the patients with PWS had the intermediate or poor metabolizing phenotype of *CYP2D6*, which could impact efficacy and tolerability of many of the psychotropic drugs used in treating patients with PWS.

**Figure 5:**
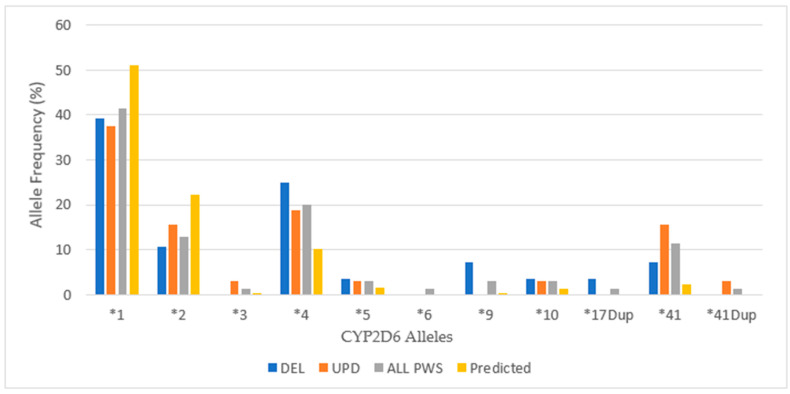
Cytochrome 2D6 allele frequencies among a referred cohort of patients with PWS DEL (*n =* 14), UPD (*n =* 16) and combined cohort (ALL PWS, *n =* 35) compared to predicted, American normative data [http://www.pharmgkb.org/page/cyp2d6RefMaterials].

When considering the PWS genetic subtypes, there were fewer extensive metabolizers, more intermediate metabolizers, and more poor metabolizers among those with DEL compared to the normative American population, and all values were statistically significant (*p* < 0.05). Among those with UPD, the ratio of EM:IM was roughly the same as in the normative American population, although the number of poor metabolizers was actually greater than among DEL, and both PM values were statistically significant (*p* < 0.05). Figure 5 displays the allelic distribution and frequencies of *CYP2D6* polymorphisms among PWS genetic subtypes in our referred cohort.

**Figure 6:**
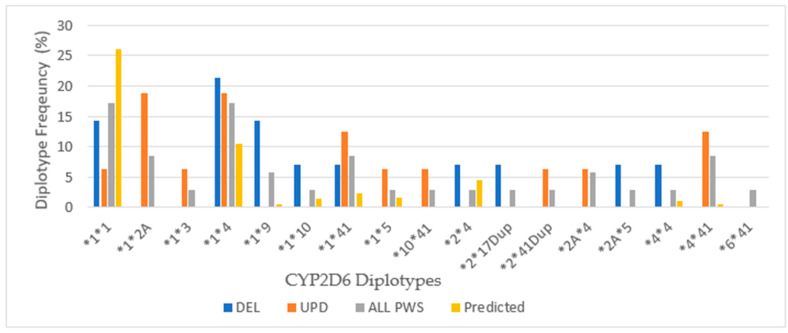
Cytochrome 2D6 diplotype frequencies among PWS DEL (*n =* 14), UPD (*n =* 16) and combined cohort (ALL PWS, *n =* 35) compared to predicted, American normative data [http://www.pharmgkb.org/page/cyp2d6RefMaterials].

In our cohort of patients, there is a lesser frequency than predicted for the most common CYP2D6 alleles *1 and *2 (both convey normal activity) and an increased frequency of alleles *4 (inactive) and *41 (reduced function). Further, the distribution of alleles includes others with a lower frequency of occurrence that are inactive or have reduced function. Subtle differences were noted in the number of alleles between the PWS genetic subtypes, but the DEL group had a higher frequency of *4 alleles, and the UPD group had a greater number of *41 alleles. See Figure 6 for *CYP2D6* diplotypes.

Among the total cohort of PWS, the wild type diplotype *1*1 occurs at a reduced rate, roughly two-thirds of the American normative population, but nearly equal to the frequency of the *1*4 diplotype, which codes for decreased activity, and exceeds the normative frequency by more than one-third. The most frequent CYP2D6 diplotypes among the DEL subtype are *1*4 and *1*9, both of which have decreased activity predicting intermediate metabolizer phenotype. The frequency of *1*1, which is the extensive metabolizing wild type, is equal in frequency to *1*9, which has decreased activity. These frequencies explain the predominance of intermediate metabolizer status among DEL. Among the UPD subtype, the highest frequencies are *1*2A (extensive metabolizing) and *1*4 (intermediate metabolizing), and the next most frequent are *1*41 (extensive metabolizing) and *4*41 (poor metabolizing), explaining the 9EM:4IM:3PM ratio of metabolizer phenotypes.

Because CYP2D6 metabolizes many antidepressants and antipsychotics often prescribed in PWS, it is not a surprise that our cohort of patients referred for treatment has alleles with reduced function contributing to decreased efficacy or adverse effects; conversely, they may respond to a lower dose [29,30]. Medications (in alphabetical order) most commonly used in PWS that are substrates of CYP2D6 include amphetamines, aripiprazole, bupropion, citalopram, clonidine, diphenhydramine, escitalopram, fluoxetine, fluvoxamine, haloperidol, olanzapine, quetiapine, risperidone, sertraline, trazadone and ziprasidone [31]. The CYP2D6 enzyme activity in our cohort showed a greater number of patients than predicted with poor metabolizer status among both PWS genetic subtypes, which could impact approximately 25% of all medications and 60–70% of behavioral/psychiatric prescribed drugs as discussed by Butler [18].

### 3.3. CYP2B6

**Figure 7:**
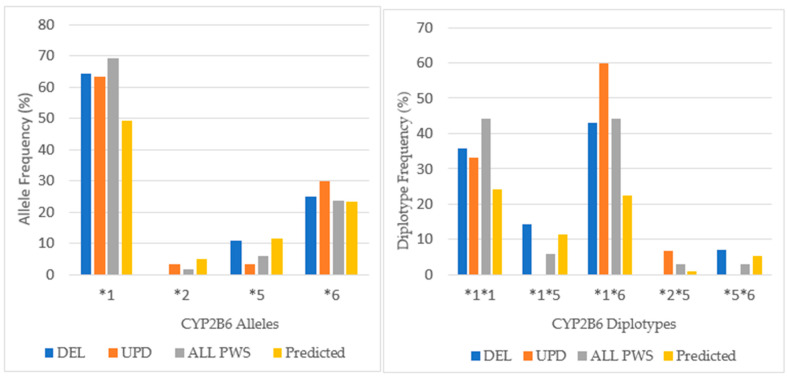
Cytochrome *2B6* allele frequency and diplotype distribution in a referred cohort with PWS DEL (*n =* 14), UPD (*n =* 15) and combined cohort (ALL PWS, *n =* 34) compared to the predicted, European normative data [http://www.pharmgkb.org/page/cyp2b6RefMaterials].

Among our total PWS cohort, 53% had the extensive metabolizer phenotype compared to 43% in a normative European population [www.pharmgkb.org/page/cyp2b6RefMaterials]. The chi-square test was not significant (*p* > 0.05). Intermediate metabolizers were found in 47% of the patients with PWS compared with 39% in the European population, but again, the chi-square value was not significant (*p* > 0.05). There were no poor metabolizers among our patients with PWS. The intermediate metabolizing phenotype predominated among the UPD cohort, whereas the intermediate and extensive metabolizing phenotypes were equal among DEL. These results were not statistically significant by chi-square test (*p* > 0.05). The distribution of alleles for *CYP2B6* is shown in Figure 7.

There were 4 alleles identified in our cohort with a greater expression of the wild type allele (CYP2B6*1) that confers normal activity in the combined cohort (69%) compared to the normative European population (47%). In the combined cohort the expression of *6, an allele associated with decreased function, is nearly equal to the normative population. Comparing DEL and UPD genetic subtypes, there is a greater expression of *6 allele among the UPD cohort.

Differences in the distribution of diplotypes are noted among the PWS genetic subtypes. Among both DEL and UPD, the frequency of the CYP2B6*1*6 diplotype, which is associated with the intermediate phenotype, is expressed more frequently than the *1*1 diplotype, which has the extensive metabolizer phenotype. In the UPD cohort, *1*6 is expressed almost twice as frequently as *1*1, and this explains the greater number of intermediate metabolizers among the UPD group. The diplotype CYP2B6*1*5, which is associated with normal function, occurs more frequently among DEL than the normative population, but is not found at all among UPD.

Among the total cohort of PWS, both the *1*1 and *1*6 diplotypes are expressed at nearly twice the frequency of the European normative population. This explains the distribution of phenotypic activity among our cohort where the extensive and intermediate metabolizer status are nearly equal. These results would suggest caution when prescribing medications metabolized by CYP2B6, such as bupropion and sertraline among the UPD group.

### 3.4. CYP2C19

**Figure 8:**
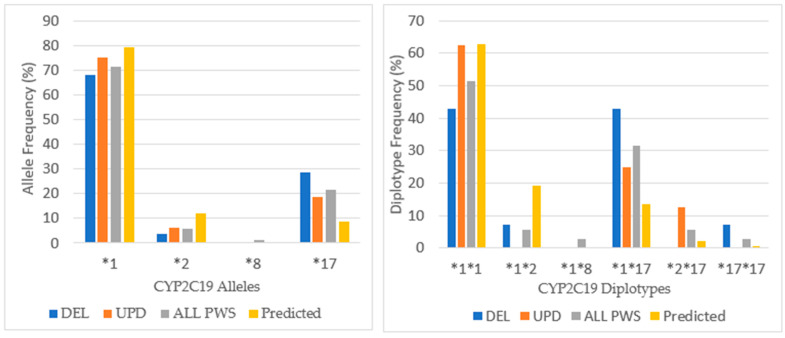
*CYP2C19* allele frequency and distribution of diplotypes in PWS DEL (*n =* 14), UPD (*n =* 16) and combined cohort (ALL PWS, *n =* 35) compared to the predicted normative American data [http://www.pharmgkb.org/page/cyp2c19RefMaterials].

Eighty-six percent of the PWS combined cohort had the extensive metabolizer phenotype of CYP2C19, and this is higher than expected from American normative data (76.4%) but not significantly different by chi-square test (*p* > 0.05). Less than 10% in the current study were intermediate metabolizers compared to 21.4% for the American population, which again was not significant by chi-square (*p* > 0.05). Ultra-rapid metabolizers were seen in 5.7% of the patients with PWS compared to 0.7% for the American data, which was not significant by chi-square (*p* > 0.05). There were no poor metabolizers among the current cohort compared to 3.2% in the Vanderbilt survey. For CYPC19, there were PWS subtype group differences. Among those with deletion, there were more ultra-rapid metabolizers compared to the normative population (*p* < 0.05). The UPD cohort displayed 100% extensive metabolizer phenotype and no intermediate metabolizers, and both results were statistically significant (*p* < 0.05) compared to the normative population of 76.4% and 21.4%, respectively. The frequency of alleles and diplotypes are compared with normative American data in Figure 8.

Among our cohort of referred patients with PWS, there is a lesser frequency of the most common *CYP2C19* alleles *1 and *2 that have normal function and an increased frequency of *17 allele, which is associated with increased function; there was one person in the PWS Unspecified diagnostic group with *8 allele (inactive). Differences were noted between the PWS genetic subtypes with the *17 allele being highest among DEL. The allelic frequency of *17 in our combined cohort was more than twice that predicted in the American population (8.6%), and three times higher among the DEL. This is the reason for the increased frequency of ultra-rapid metabolizers among DEL and among the total cohort as well.

The frequency of the normal functioning diplotype *1*1 is less than predicted by normative data except among the UPD group. Among the UPD cohort, only extensive metabolizers were found. The number with increased function *1*17 is more frequent due to the presence of *17 allele, especially among DEL. Also, the ultra-rapid functioning diplotype *17*17 is noted among DEL but not UPD. Overall, the extensive metabolizer phenotype of CYP2C19 prevails in our PWS cohort largely due to the increased expression of the *1 and *17 alleles.

Many psychotropic medications used in patients with PWS are substrates for CYP2C19, including (but not limited to) citalopram, clomipramine, doxepin, escitalopram, fluoxetine, imipramine, and sertraline [32]. It is inferred that these medications would have been well tolerated by most of our referred cohort. However, gonadal steroids (estradiol and testosterone), which are replaced commonly in PWS due to delayed or absent puberty, are substrates for CYP2C19, indicating a potential for drug interactions. This is discussed more fully in Section 4.1 and Section 4.2.

### 3.5. CYP2C9

The prevailing phenotype of CYP2C9 in our cohort was extensive metabolizing (61.8%), which was decreased in comparison to American normative data (83.2%) and was significantly different by chi-square test, *p* < 0.05. The intermediate metabolizing phenotype was found in 35% of the combined cohort, and this is twice the number predicted in the American population (16.4%) and significantly different also by chi-square test (*p* < 0.05). For CYP2C9 the DEL cohort was 76.9% extensive metabolizer and 23.1% for intermediate, remarkably similar to American normative data at 83.2% and 16.4%, respectively. But for the UPD cohort, extensive metabolizer was 56.3%, intermediate 37.5%, and poor 6.3%; all of these values were statistically significant by chi-square (*p* < 0.05).

**Figure 9:**
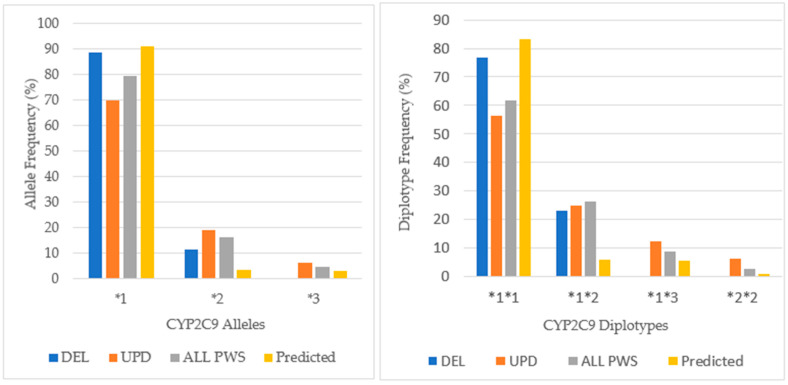
*CYP2C9* allele and diplotype frequencies in PWS DEL (*n =* 13), UPD (*n =* 16) and combined cohort (ALL PWS, *n =* 34) compared to American normative, predicted data [http://www.pharmgkb.org/page/cyp2c9RefMaterials].

Among our cohort of patients with PWS, the distribution of alleles in Figure 9 shows a predominance of CYP2C9*1, which confers normal activity at a lesser frequency than in the normative American population. There is a greater occurrence of the CYP2C9*2 allele (16.2% of ALL PWS) compared to the frequency in the American population (3.3%) with greatest prevalence among UPD (18.8%). Alleles *2 and *3 have decreased activity, and there were no CYP2C9*3 alleles among DEL. Among the UPD cohort, the frequency of CYP2C9*1 allele was less than DEL, and the CYP2C9*2 allele frequency was nearly twice that found among DEL.

The CYP2C9*1*1 diplotype that confers the normal phenotype has the greatest frequency across all PWS genetic subtypes, although it occurs more frequently among DEL than UPD. All other pairs are intermediate metabolizing, and there is a greater proportion of these pairs in UPD compared to DEL. This likely contributes to the greater number of intermediate metabolizers among UPD, twice as many as DEL. More than half of patients with UPD in this cohort may have required dosage adjustment for drugs such as amitriptyline, fluoxetine, and sertraline especially when used with concurrent oral contraceptives, methylphenidate, modafinil and omeprazole [31].

### 3.6. CYP3A4

**Figure 10:**
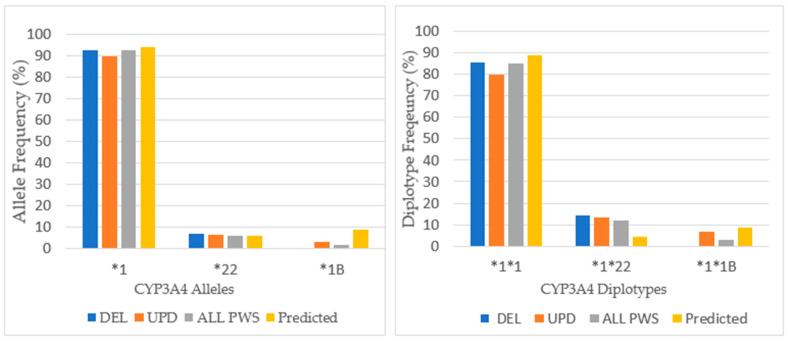
*CYP3A4* allele and diplotype frequencies among PWS DEL (*n =* 14), UPD (*n =* 15), and combined cohort (ALL PWS, *n =* 34) compared to Caucasian normative, predicted data [33,34].

In the admixed American population, 97.3% had the extensive metabolizing phenotype of CYP3A4 [20]. Across all PWS genetic subtypes the predominate phenotype of *CYP3A4* was extensive metabolizing; there were only 5 patients who had intermediate metabolizing status. *CYP3A4* has over 30 polymorphisms, most of which occur at low allelic frequencies. The distribution of alleles and diplotypes is found in Figure 10.

There were only three alleles present in the analysis of CYP3A4: *1A, *22, and *1B across PWS genetic subtypes in our cohort of referred patients. The wildtype gene, CYP3A4*1A, was the major allele expressed with a frequency of 92.6% that compared favorably with Caucasian population norms of 92.1% [33]. CYP3A4*22 is a reduced function allele. The allelic frequency for CYP3A4*22 in this study was 5.9%, and this was consistent with population norms of 5–7% [35]. CYP3A4*1B was expressed only among the PWS UPD subtype at a frequency of 3.3%, which is less than allele frequencies of 7.9% reported among Caucasians [33]. This value was not statistically significant by chi-square, *p* > 0.05. CYP3A4*1B has been associated with patients who have cancer, and recently Swiechowski et al. found that compared to controls, patients suffering from recurrent major depressive disorder were more likely to have the heterozygous (AG) CYP3A4*1B genotype [36]. In this study comparing 102 patients with 90 controls, the G allele frequency was higher among patients than controls, and those with the homogeneous (GG) genotype, although fewer in number, reported an earlier age of onset of depression. Even though CYP3A4 is involved in the metabolism of many antidepressant medications, such as tricyclics, SSRIs, SNRIs, and mirtazapine, the phenotypic results did not support any differences in metabolism [36].

The distribution of diplotypes finds that the frequency of the wildtype alleles *1A*1A predominates across all PWS genetic subtypes at nearly typical frequency. The occurrence of the reduced function *1*22 diplotype is greater than predicted among all PWS genetic subtypes [34].

### 3.7. CYP1A2

For *CYP1A2*, the frequency of ultra-rapid metabolizing phenotype exceeded the extensive metabolizing among the total cohort, and this most likely reflects the influence of the 2:1 frequency of ultra-rapid metabolizers to extensive metabolizers among patients with UPD. Further there is a low frequency of intermediate metabolizers, and there are no poor metabolizers. Compared to other cytochromes, *CYP1A2* appears to be unique in the number of polymorphisms, the variety of inherited allelic combinations, and the capacity for induction from medicinal, dietary, gender, hormonal, and lifestyle factors [14].

Across all PWS subtypes, there was an increase in the number of ultrarapid metabolizers, especially among UPD (*p* < 0.05), and there were no poor metabolizers across the total PWS cohort compared to the normative population (*p* < 0.05). A significant difference was noted by chi-square test (*p* < 0.05) when comparing the frequency of CYP1A2 IM metabolic phenotype to the normative population (54%) in our patients with DEL (0%) and UPD (6.25%) [27].

**Figure 11:**
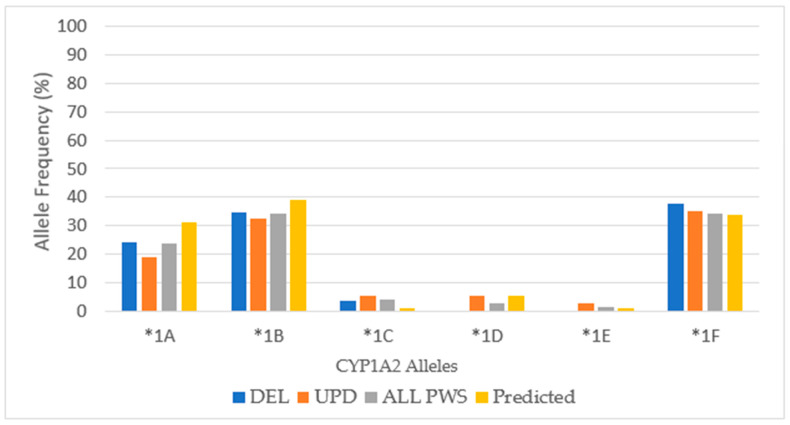
*CYP1A2* allelic frequencies in PWS DEL (*n =* 14), UPD (*n =* 16) and combined cohort (ALL PWS, *n =* 35) compared to normative Caucasian predicted values [37,38].

There were six alleles present in the analysis of *CYP1A2* in our cohort (*1A, *1B, *1C, *1D, *1E, and *1F) and their frequencies among each genetic subtype are presented in Figure 11 and compared to predicted values among Caucasians [37,38].

CYP1A2*1A and *1B have normal function and CYP1A2*1C has decreased function. Both CYP1A2*1D and CYP1A2*1F are inducible. For example, both CYP1A2 *1A and *1F express the extensive metabolizer phenotype in the absence of an inducer, but in the presence of tobacco smoke, insulin, modafinil, nafcillin, omeprazole or cruciferous vegetables, the hyper-inducible (HI) phenotype is expressed by *1F [38]. Among the pharmacogenetic results from the Genelex company, the *1F haplotype was identified as having the HI phenotype. To achieve consistency across the data set for *CYP1A2* in Table 1, we combined the results of HI into EM.

The gene for *CYP1A2* is located on chromosome 15 (15q24.1) outside the PWS critical region. Therefore, it is possible that in the PWS patient with maternal uniparental disomy 15 (UPD), the *CYP1A2* alleles may be identical. The clinical relevance of this depends upon the phenotypic activity determined by the *CYP1A2* alleles seen in the mother. Almost all people have 2 copies of the *CYP1A2* gene [38]. Among our cohort of patients with UPD, there were 3 duplications of alleles, whereas cohorts with DEL and PWS Unspecified had only one. Also, there were complex genotypes of *CYP1A2*, some with as many as 4 alleles. Clinical implications for treatment with these *CYP1A2* findings in PWS are discussed in Section 4.1 and Section 4.2.

## 4. Discussion

The use of pharmacogenomic testing is now commonly obtained, particularly after an untoward result of a medication treatment trial. It provides guidance for medication selection and dosing. There are several studies suggesting that genotypically informed medication selection for treatment of depression increases response and remission rates, decreases adverse effects, and guides the use of adjunctive medications in challenging cases [39,40].

Knowledge of the pharmacogenetic phenotype can inform time parameters of treatment response. Ultra-rapid metabolizers are more likely to have a quicker, positive clinical response to antidepressant medication with several studies suggesting an increased risk of rehospitalization and emergency room visits [41]. In a study performed at the Institute of Living, those patients with the CYP2D6 ultra-rapid metabolizing genotype were more likely to be discharged early and to have at least one readmission within the month after discharge [42]. On the other hand, those patients with intermediate or slow metabolizing genotypes were likely to take a longer time to achieve a clinical treatment response, a longer duration of hospital stay, and a decreased likelihood of readmission in the 30 days after discharge [42]. In another study, intermediate metabolizers were more likely to have adverse effects to medications metabolized by CYP2D6 than extensive metabolizers at comparable doses [43].

Knowledge of the pharmacogenetic phenotype can inform dose response parameters [44]. For example, if a person has the poor metabolizing phenotype of CYP2D6, which metabolizes paroxetine exclusively, a typical starting dose of medication may produce a therapeutic effect, and according to Kirchheiner et al. the dose required for remission may be 65% of the standard dose [30]. If the person is an intermediate metabolizer, they may tolerate low to moderate doses of medication, but as the dose is titrated, their metabolic capacity may be exceeded resulting in side effects [45]. On the other hand, if a person is an ultra-rapid metabolizer, the half-life of the medication will be reduced and the patient may experience unpleasant withdrawal symptoms during the day [29], such as headache and gastrointestinal upset without fever, mood instability, and what is been described as the perception of a lightning bolts radiating down the arms. With this ultra-rapid genotype, these symptoms can be addressed by increasing the schedule of administration of medication across the day. As a result, and following the standard of care, the patient may require a higher total daily dose, e.g., for paroxetine, 135% of the standard dose, than typically recommended by the FDA for that medication and condition as discussed in Kirchheiner et al. [30].

Knowledge of the pharmacogenetic phenotypes can inform other parameters of medication response, including potential adverse effects. If a medication requires conversion to an active metabolite by the cytochrome enzyme system, the phenotype will determine the rate of activation. The best example of this is codeine, which requires CYP2D6 for conversion to the active metabolite morphine. Poor metabolizers of CYP2D6 are at risk for a poor analgesic response, as ultra-rapid metabolizers are at risk for toxicity [29]. Another example is risperidone, one of the most widely prescribed second generation antipsychotic medications among children and adolescents, including with PWS, with known efficacy and adverse effects, especially among those with autism spectrum disorder (ASD) and intellectual and developmental disabilities (IDD) [46]. Risperidone is primarily metabolized by CYP2D6 into another active metabolite, paliperidone, which is linearly related to serum prolactin level. Individuals with the ultra-rapid metabolizing phenotype of CYP2D6 may be more likely to display hyperprolactinemia [47]. In a study of 257 children and adolescents who received risperidone, 76 experienced a variety of adverse effects, and these were more commonly seen in the poor or intermediate metabolizing phenotype of CYP2D6 [48].

Knowledge of potential drug interactions can inform treatment and minimize adverse effects. Drug interactions can occur when using prescribed medications as well as over the counter (OTC) agents and nutraceuticals (herbs, supplements, or vitamins) [14].

### 4.1. Drug–Drug Interactions

Drug-drug interactions are a common cause of adverse events or failed treatment efficacy. Checking for potential drug interactions can be accomplished by examining the pharmacokinetic pathways for medication metabolism at https://www.pharmgkb.org/pathways and https://drug-interactions.medicine.iu.edu [32]. The Flockhart table is updated frequently and delineates the pharmacogenetic action of specific drugs that use the cytochrome P450 enzyme system. Drugs are delineated as substrates, inhibitors, or inducers for one or more cytochrome P450 enzymes. Medications administered concurrently that are substrates for the same cytochrome enzyme overwhelm the metabolic capacity of the cytochrome and interfere with efficacy or may result in toxicity [45]. If two drugs have the same affinity for an enzyme, they become competitive inhibitors in a dose related way [49]. If one of the drugs is a substrate for another cytochrome P450 enzyme with less affinity, metabolism may be shunted toward those less preferred pathways. An inducer is a drug or agent (e.g., cigarette smoking) that causes an increase in the production of the cytochrome enzyme by action at the promoter site on the gene and usually takes 1–2 weeks to occur [39,40]. An inhibitor is a drug or agent (e.g., cruciferous vegetables) that binds to the cytochrome enzyme and blocks its use; its effect is immediate and persistent for as long as the inhibitor remains in the system. Some medications act as both substrate and inhibitor (e.g., fluoxetine with CYP2D6), or substrate and inducer (e.g., carbamazepine with CYP3A4) [35].

Phenoconversion describes the process whereby a given CYP phenotype, inferred from genotype, is functionally converted to a higher or lower metabolic status on the basis of drug-drug interactions or the influence of non-genetic factors [50]. The resulting phenotype carries the designation of phenocopy because it imitates a different metabolic status. Phenoconversion is more likely to occur in drug-drug interactions when a substrate has a high affinity for a single CYP. For example, clozapine and olanzapine are selectively metabolized by CYP1A2; in the presence of cigarette smoking, phenoconversion to a higher metabolizing phenotype results in the reduction of serum drug levels by 20% [43]. In the case of aripiprazole, concurrent administration of a CYP2D6 inhibitor, such as bupropion or sertraline, can convert the 2D6 extensive metabolizing status downward, driving serum drug levels upward by 20–50%, precipitating adverse effects [51]. In another example, ratios of metabolic phenotypes that were inferred from genotype across cytochromes 2C9, 2C19, 2D6 and 3A4 were converted to a lower status in the presence of inflammation, as reviewed by Klomp et al. [50].

Table 2 itemizes psychotropic and other prescribed medications, OTC agents, nutraceuticals, and dietary/lifestyle factors that are substrates, inhibitors, or inducers of cytochrome P450 enzymes. Most of the items in this table were generated from clinical experience with persons who have PWS and by referencing Flockhart [32]. This table does not constitute a complete list of medications, nor is it a compendium of recommended therapeutic agents for patients with PWS.

Drug–drug interactions may occur with prescribed medications as well as OTC agents and nutraceuticals. OTC antihistamines, such as loratadine (substrate of CYP3A4), may interfere with metabolism of some antidepressants and antibiotics [52]. Chlorpheniramine and dextromethorphan, OTC agents used for common cold symptoms, are substrates for CYP2D6 [32]. Competition at the site of the enzyme receptor can displace risperidone and potentiate its efficacy producing increased sedation or extrapyramidal side effects such as akathisia. The anti-acid medication ranitidine, which was recently removed from the market, is a substrate for CYP2D6 and competes with several atypical antipsychotic medications, including risperidone [18]. Toxic side effects have been reported, including incapacitating sedation that was misdiagnosed as dementia in a patient with PWS. Similarly, omeprazole is a substrate for CYP2C19 and CYP2C9 and inhibits metabolism of many antidepressants contributing to the potential toxicity. Acetaminophen, a substrate for CYP1A2, and ibuprofen, a substrate for CYP2C9, may affect efficacy of psychotropic medications administered concurrently [38]. Melatonin, a substrate of CYP1A2, is commonly used for treatment of insomnia, and treatment with fluvoxamine results in increased serum melatonin [53].

Herbal or plant-based agents may also interact with psychotropic medications. Ginkgo biloba, used to treat memory and dyskinesias, has been associated with increased risk of hemorrhage in combination with SSRIs or SNRIs [54]. Ginseng, used to increase energy, stamina and well-being, has been associated with serotonin syndrome in combination with SSRIs or SNRIs, and ventricular arrythmias in combination with haloperidol [54]. Goldenseal, used for upper respiratory and gastrointestinal tract symptoms, is a potent inhibitor of CYP3A and CYP2D6 [53]. Milk thistle, used for diabetes or liver disease, has resulted in pancreatitis when co-administered with haloperidol or risperidone, and hepatotoxicity may occur with aripiprazole [54]. St. John’s Wort, a natural occurring antidepressant, interferes with the efficacy of many pharmaceuticals due to induction of cytochromes 1A2, 2C9 and 3A4 [55]. Both cannabis and cannabidiol are substrates for CYP2C19 and CYP3A4 and inhibitors of CYP2D6 and CYPC9; also, cannabis is a substrate for CYP2C9, and cannabidiol is an inhibitor of CYP2C19 [56]. Their concurrent use with fluoxetine, a substrate for CYP2D6, CYP2C9, and CYP2C19 and an inhibitor of CYP2C9 and CYP2C19, can be expected to produce a complex drug interaction that may result in mood activation. Because each of these agents has a relatively long half-life, the onset of any clinically relevant interaction may be delayed, and once it is identified, the discontinuation of one of the medications will take a while to clear the body, so unpleasant symptoms may linger. The persistence of mood and behavioral symptoms may lead to the diagnosis of a co-morbid psychiatric condition or may require management with yet another psychotropic medication.

Another common drug-drug interaction is seen with SSRIs (citalopram, escitalopram and sometimes sertraline) and estradiol and/or progesterone (taken for hormone therapy, oral contraception, or occurring naturally with monthly menstrual cycles), which are substrates for CYP2C19. A woman receiving sertraline for depression who experiences premenstrual dysphoria may benefit from a transient dose increase during the luteal phase as progesterone levels increase [57,58]. Because puberty is often delayed or absent in PWS, hormone therapy is usually initiated during the age of typical adolescence. If SSRIs had been started previously, the addition of estradiol or a combination pill may compete with CYP2C19 metabolism altering serum levels and potentially precipitating mood and behavioral difficulties requiring a dose adjustment. Hormone therapy is likely to continue into adulthood in PWS to address lifelong osteopenia and osteoporosis.

The frequency of polypharmacy use identified in the NIH PWS Registry suggests that drug interactions, like the ones identified above, may be commonplace. Knowledge of pharmacogenetics may inform dose parameters as well as potential drug interactions. For patients with PWS, like others with intellectual and developmental disabilities, it is always advisable to try one medication at a time, or to add another medication after a new behavioral baseline has been established. This applies for hormone therapies and use of nutraceuticals as well.

### 4.2. Specific Relevance of Cytochrome P450 Enzyme System to PWS

There are factors that can change the phenotypic expression of the cytochrome P450 genes that are particularly relevant to PWS. *CYP3A4,5* is sexually dimorphic due to gonadal steroid effects on gene activity. There is evidence to suggest that growth hormone is a modulator of this gender specificity of function. Sinues and colleagues [59] examined CYP3A enzyme activity in 35 unrelated growth hormone deficient children (ages 2.9–13.1 years) both at baseline and after growth hormone replacement. At baseline, the level of activity of CYP3A was elevated compared to controls in a non-sex dependent manner. Then, after growth hormone replacement for 30 days, CYP3A activity was reduced to normal range in males but was unchanged in females. This typical sexual dimorphic level of activity for the CYP3A enzyme impacts serum levels of testosterone, gonadal steroids, as well as psychotropic drugs. This has special relevance for individuals with PWS who are likely to be growth hormone deficient and require growth hormone replacement as well as gonadal steroid therapy [1].

The activity of CYP1A2 has gender specificity also, with lower metabolic function in women than in men. [60,61]. The primary function of CYP1A2 is to purge potential environmental toxins, e.g., heterocyclic amines, and polycyclic hydrocarbons, that may act as carcinogens from the body. Pharmacogenetic studies have explored polymorphisms of these alleles as predisposing factors to the incidence of cancers of the urinary tract, colon, and rectum [37]. CYP1A2 is inhibited by estradiol, oral contraceptives, some antibiotics (ciprofloxacin and levofloxacin), and other drugs (fluvoxamine, celecoxib, and amiodarone). Polymorphisms of *CYP1A2* have been explored as predisposing factors for psychiatric illness [62]. Yenilmez et al. found that the allelic frequency of CYP1A2*1F among those with bipolar disorder was 25.5% and among those with schizophrenia, it was 69.4%, which is twice that predicted for normative population frequencies [62]. Because many psychotropic medications used for treating these conditions are also substrates for CYP1A2 (e.g., olanzapine, clozapine, haloperidol, fluvoxamine, duloxetine, tricyclic antidepressants, and clomipramine) [18], their metabolism is subject to the effects of induction, which reduces serum concentration and can interfere with treatment efficacy. Because of hypogonadism and delayed puberty in PWS, females are often prescribed estradiol or estrogen/progesterone combinations found in oral contraceptives, both of which inhibit gene expression and activity of CYP1A2. This inhibition may result in increased drug levels of substrates of CYP1A2 with potential toxicity [32,63].

*CYP1A2* is highly inducible by dietary factors and lifestyle considerations [18]. Caffeine is a well-recognized substrate of CYP1A2, and it is used to determine enzyme activity [37]. The CYP1A2*1D and CYP1A2*1F alleles predict the ultra-rapid metabolizing phenotype when the enzymes are induced. Common inducers are tobacco and cannabis smoke, chargrilled meats, cruciferous vegetables (e.g., brussels sprout, broccoli, cauliflower, cabbage, radish, rocket, watercress, and wasabi), insulin, modafinil, nafcillin, omeprazole, and carbamazepine [14,38]. Xie at al. reviewed the magnitude of induction effects, with 3.5-fold increase in smokers vs. non-smokers and 25% increase with broccoli containing diets [63]. The inducibility of CYP1A2 by diet is particularly important to persons with PWS, many of whom follow the Red Yellow Green Diet that contains a high percentage of cruciferous vegetables that are low in calories and high in fiber [64]. Further, many persons with PWS are prescribed modafinil (CYP1A2 inhibitor) for excessive daytime sleepiness. Cigarette smoking and overuse of caffeinated beverages (CYP1A2 inducers) are not uncommon among adults with PWS. Severe skin picking may result in cellulitis that requires systemic antibiotic treatment (CYP1A2 inhibitors) [65]. Gastroesophageal reflux is common in PWS and often treated with proton pump inhibitors such as omeprazole (CYP1A2 inducer) [65,66].

### 4.3. Non-Genetic Factors Affecting Drug Pharmacokinetics in PWS

There are non-genetic pharmacokinetic factors determining dose response efficacy, and these factors are especially important in the pediatric population. In this report, the cytochrome system pertains to those enzymes in the liver responsible for metabolizing medications. Overall, the metabolic activity of the liver is increased during the developmental years before puberty such that metabolic clearance in children is twice as fast as in adolescence [29]. Therapeutic doses of medications metabolized by CYP1A2, CYP2C9 and CYP3A4 are much higher in children than compared to adults, and the bioavailability of medications that require first pass metabolism for activation is decreased [56]. Increased metabolism suggests a shorter half-life and indicates that the medication should be dosed more frequently through the day. These developmental changes are not seen with CYP2D6 or CYP2C19 [61].

Diseases or nutritional status that affect the liver function will impair metabolism at any age, such as steatohepatitis. In a study by Li et al., CYP2C19 activity was decreased by as much as 80% in adolescents with steatohepatitis (not steatosis) while activity of CYP1A2, CYP2C9 and CYP3A4 was unaltered, unlike the pattern of change in adults resulting in decreased activity of CYP2C19, CYP1A2 and CYP3A4, and increased activity of CYP2C9 [67].

There are other factors affecting drug metabolism such as body weight or body surface area that determine the daily dosage of medication before puberty. As reviewed by Li et al., obesity is associated with an increase in CYP1A2, CYP2C9, CYP2C19, and CYP2D6 activity in children, and a decrease in CYP1A2 and CYP3A4 activity in adults [67]. Body composition is also important, because lipophilic drugs accumulate in adipose tissue and prolong rate of excretion. The mode of delivery of medication (intramuscular, subcutaneous, or intravenous injection; intranasal, sublingual, or rectal administration; or topical application) can affect speed of onset of action, effectiveness on the target symptom, and extent of systemic side effects. Lifestyle factors can also affect cytochrome function, e.g., consumption of chargrilled foods and cruciferous vegetables induces CYP1A2 and CYP3A4 activity; grapefruit juice inhibits CYP3A4; caffeinated beverages inhibit CYP1A2, and smoking cigarettes or cannabis will induce CYP1A2 [60]. Stomach acidity can also affect absorption, and urine pH can affect excretion, especially with amphetamines [68].

Finally, compliance is the most important variable in determining medication efficacy. In general, the more frequently a medication must be administered, the higher the likelihood that doses will be missed. In the experience of PWS experts who administer care to patients with PWS, it is always preferable to start a regular acting medication first to ascertain dose and side effects before converting to a sustained release form (J.F, personal communication; [69]).

### 4.4. Limitations and Future Directions

The data presented in this clinical case series were obtained from approved commercial laboratories. Not all commercial pharmacogenomic platforms test the same cytochrome P450 genes and polymorphisms. Companies select the polymorphisms to test based on the patient population, the frequency of polymorphisms, and the clinical relevance for a particular field of medicine, e.g., oncology or psychiatry. Also, the clinical interpretation of testing results may differ. The genetic data obtained may be the same, e.g., both the number and type of alleles, but the designated phenotype is determined by combining results from several genes. The algorithm for this combinatorial approach is proprietary [19].

The idea for reporting this clinical case series was conceptualized after the initial collection of data within our respective clinical practices. The patients who received pharmacogenomic testing met medical necessity criteria set forth by the companies based on insurance coverage, including failure of at least one psychiatric medication. Therefore, our cohort represented a biased population of PWS patients who might be expected to have some alterations of cytochrome P450 function. In this case report, there is no PWS control group for comparison. In addition, the information available for each patient came from three different clinical centers, was subject to record availability (age, gender, ethnicity), and was deidentified prior to data collation and analysis. A history of psychotropic medication use as well as family history of psychiatric conditions and medication response was not discoverable. Also, non-genetic, environmental factors that may influence how these patients metabolize psychotropic medications were not known. Our data was limited to the genetic polymorphisms of selected cytochrome P450 enzymes, our interpretation of these findings, and the PWS genetic subtype of the patient. We have provided a descriptive analysis of these results.

This data set was limited in number and did not reach sufficient power for PWS-subtype analysis in all cases. In particular, the numbers were too small to undertake analysis of gene allele frequencies via Hardy-Weinberg equilibrium studies. None the less, we found statistically significant differences in the metabolizing status of cytochromes 2D6, 2C9, 2C19, 3A4 and 1A2. The results from our cohort may not reflect the general PWS patient population, as the patients referred to our specialty centers are often those who have complicated health/behavior/psychiatric concerns and have failed previous medication management. Further, our PWS cohort consisted of more patients with UPD (46%), which is higher than predicted based on studies showing that 35% of PWS individuals have this genetic subtype [1,2,3]. This may reflect that those with UPD may have more behavioral/psychiatric problems and are more likely to seek mental health intervention. In the future, a larger study of at least 100 subjects from the general population of persons with PWS would provide sufficient statistical power to correlate pharmacogenetic data with factors of age, gender, ethnicity, and family psychiatric history. Also, a larger study would offer greater insight and guidance into medication management in this rare population often presenting with significant psychiatric needs. Until a larger study is performed, the authors realize that the interpretation and generalization of these results is limited to our cohort of referred patients.

Except for *CYP1A2*, which is located on Ch 15, we cannot explain the frequency of phenotypic differences in the cytochrome P450 system noted in this case series. UPD status due to maternal isodisomy 15 may have altered *CYP1A2* allele frequencies in our referred cohort [2]. The most common etiology for CYP polymorphism is familial, which includes variations associated with ethnicity. The ethnicity of our 35 patients was deidentified, and we did not have access to familial psychiatric history. However, the NIH PWS Registry enrolled patients from PWS clinics at 4 sites in the USA, and the racial/ethnic diversity of these 355 patients was minimal with 93% Caucasian. As such, our phenotypic comparisons and statistical correlations were all based on normative Caucasian populations. It is highly unlikely that our pharmacogenetic findings in this PWS cohort were due to ethnic variability.

Finally, one of the criteria for obtaining pharmacogenetic testing includes failure to respond to current treatment with psychotropic medications. The PWS phenotype is a complicated mix of anxiety, stress sensitivity, mood lability, and disruptive behavior. Clinicians assume that these symptoms will respond to psychotropic medication. But it is possible that these symptoms require environmental, psychological, or behavioral management strategies for optimal response. Therefore, treatment failure with psychotropic medications alone cannot necessarily be attributed to polymorphisms of the cytochrome enzyme system.

## 5. Conclusions

This clinical case series of patients with known PWS subtype is the largest reported cohort to receive commercial pharmacogenomic testing. At first glance, the results from our combined cohort suggest that our patients with PWS had a wide array of alleles that were distributed in a manner consistent with natural variation. However, these patients were referred for evaluation and treatment, and not surprisingly, group differences were noted when comparing the results of PWS testing with normative population data. For example, 48.6% of the combined PWS cohort (*n =* 35) were extensive metabolizers of CYP2D6 compared to the typical population frequency of 63.6% among Americans. There were 36% intermediate metabolizers and 17.1% poor metabolizers compared to 23.6% and 2.2% respectively among Americans, which means that more than half of our PWS cohort had reduced function of CYP2D6. Further, there were differences between the molecular classes of PWS with the deletion subtype having fewer extensive metabolizers and more intermediate metabolizers; both DEL and UPD subtypes had more poor metabolizers compared to the normative American population. These results suggest that increased vigilance is required by clinicians for dose selection and speed of titration of psychotropic medications used in patients with PWS that are substrates for CYP2D6, such as aripiprazole, bupropion, chlorpromazine, citalopram, clonidine, doxepin, escitalopram, fluoxetine, haloperidol, imipramine, mirtazapine, olanzapine, quetiapine, risperidone, sertraline, trazadone, venlafaxine and ziprasidone. The clinical mantra of *start low and go slow* is recognized and indicated in the treatment of clinical symptoms among patients with PWS as well as other intellectual and developmental disabilities [45]. The increased frequency of ultra-rapid metabolizing status of CYP1A2 in PWS, especially among those with UPD, suggests that careful selection of drug dose is indicated for agents that are substrates, such as acetaminophen, benztropine, chlorpromazine, clomipramine, clozapine, duloxetine, estradiol, fluvoxamine, haloperidol, imipramine, mirtazapine, and olanzapine [32]. Further, growth hormone status and gender may play a role in pharmacokinetic parameters of patients receiving medications that are substrates for CYP3A4 primarily, such as aripiprazole, guanfacine, mirtazapine, modafinil, quetiapine, trazadone, and ziprasidone [32,69]. Also, both estradiol and testosterone are substrates for CYP3A4. Increased expression of the CYP3A4*1B allele was seen only among UPD in this case series. This allele has been associated with risk of prostate cancer, leukemia, early puberty and major depressive illness [64]. Gender may play a role in the dose selection for persons receiving bupropion or sertraline, as the number of CYP2B6 extensive metabolizers may be higher in PWS as a whole and among females with deletion subtype specifically [70]. A larger cohort of non-referred persons with PWS is needed to substantiate these preliminary pharmacogenetic findings and their applications to the clinical setting where patients with this rare disorder are treated.

In conclusion, like any medical test, pharmacogenetics testing requires that medical necessity criteria be met. These criteria include symptom severity, co-morbid illnesses, and number of failed medication treatment responses. These factors increase the likelihood that more than one medication will be used, as is often seen in PWS. Polypharmacy increases the potential for drug interactions. The pharmacogenetic data from this case series of referred PWS patients underscores the benefit of knowing the phenotypic function of the cytochrome P450 enzyme system to inform selection of psychotropic medication and dose to improve the care and treatment of those with this rare genetic condition. Although our cohort was too small to discover a unique pharmacogenetic phenotype among our patients, we did identify some statistically significant differences in phenotypic function compared to population norms and also between PWS genetic subtypes. Going forward, a larger cohort of non-referred persons with PWS must be studied to confirm these observations for widespread clinical application. Nonetheless, this data supports the use of pharmacogenetics testing in Prader-Willi syndrome.

## Figures and Tables

**Table 1:** Frequencies of CYP phenotypes in PWS cohort compared to a typical population, and substrates most likely to be affected based on drug utilization data from the NIH PWS Registry.

CYP Gene/Metabolizer Phenotype	CYP Phenotype Frequency						Substrate Frequency
	PWS Referred Cohort				Typical Population		PWS Clinic Patients
	DEL	UPD	Unspec	All PWS	Frequency/Reference		NIH PWS Registry [12,13]
***CYP2D6***	**(*n =* 14)**	**(*n =* 16)**	**(*n =* 5)**	**(*n =* 35)**	**(*n = 56,945)***		**(*n =* 265)**
EM	28.5% †	56.3%	4	48.6%	63.6%	http://www.pharmgkb.org/page/cyp2d6RefMaterials	Fluoxetine (21.9%), Risperidone (14%), Sertraline (14%), Aripiprazole (9.8%), Citalopram (8.7%), Escitalopram (5.7%), Paroxetine (4.5%), Bupropion (4.5%), Amphetamine (4.2%), Clonidine (3%), Ziprasidone (3%)
IM	57.1% *	25.0%	0	34.0%	23.6%		
PM	14.3% †	18.8% *	1	17.1% *	2.2%		
***CYP2B6***	**(*n =* 14)**	**(*n =* 15)**	**(*n =* 5)**	**(*n =* 34)**	**(*n = 56,945*)**		
EM	50%	40%	5	53%	43%	http://www.pharmgkb.org/page/cyp2B6RefMaterials	Sertraline (14%), Bupropion (4.5%)
IM	50%	60%	0	47%	39%		
***CYP2C19***	**(*n =* 14)**	**(*n =* 16)**	**(*n =* 5)**	**(*n =* 35)**	**(*n = 56,945*)**		
EM	78.6%	100% †	3	85.7%	76.4%	http://www.pharmgkb.org/page/cyp2C19RefMaterials	Fluoxetine (21.9%), Sertraline (14%), Citalopram (8.7%), Escitalopram (5.7%)
IM	7.1%	0% †	2	8.6%	21.4%		
UM	14.3% †	0%	0	5.7%	0.74%		
***CYP2C9***	**(*n =* 14)**	**(*n =* 16)**	**(*n =* 5)**	**(*n =* 35)**	**(*n = 56,945*)**		
EM	76.9%	56.2% *	2	61.8% *	83.2%	http://www.pharmgkb.org/page/cyp2C9RefMaterials	Fluoxetine (21.9%), Sertraline (14%), Valproate (6.4%)
IM	23.0%	37.5% *	3	35.3% *	16.4%		
PM	0%	6.3% †	0	2.9%	0%		
***CYP3A4***	**(*n =* 14)**	**(*n =* 15)**	**(*n =* 5)**	**(*n =* 34)**	**(*n =* 5789)**		
EM	85.7% †	80.0% †	5	85.3% *	97.3%	Zhou et al., 2017 [20]	Risperidone (14%), Sertraline (14%), Modafinil (12.8%), Aripiprazole (9.8%), Citalopram (8.7%), Clonazepam (6.8%), Escitalopram (5.7%), Bupropion (4.5%), Ziprasidone (3%)
IM	14.3% †	20.0% †	0	14.7% *	2.7%		
***CYP1A2***	**(*n =* 14)**	**(*n =* 16)**	**(*n =* 5)**	**(*n =* 35)**	**(*n =* 183)**		
UM	42.9% *	62.5% *	2	51.4% *	0%	Muscat et al., 2008 [27]	Fluvoxamine (1.1%), Haloperidol (1.1%), Thioridazine (1.1%), Olanzapine (0.4%), Chlorpromazine (0.4%), Imipramine (0.4%)
EM/HI	57.1%	31.2%	3	45.7%	37.0%		
IM	0% †	6.25% †	0	2.85%	54.0%		
PM	0%	0%	0	0% †	10.0%		

KEY: Phenotype% = within group comparison; (*) = statistical significance by chi-square, *p* < 0.05; (†) = statistical significance by chi-square, *p* < 0.05, but results may not be reliable due to small cell size. The frequency of psychotropic medications in this table is derived from the NIH PWS Registry [12,13]. Only the most frequently prescribed medications are listed for each cytochrome. This data reflects regional prescribing practices; it does not reflect treatment efficacy, nor does it constitute recommended treatment.

**Table 2:** Psychotropic medications, nutraceuticals, agents, and related cytochrome P450 enzymes.

Cytochrome	CYP1A2	CYP2B6	CYP2C19	CYP2C9	CYP2D6	CYP3A4-5
Substrate	Acetaminophen Amitriptyline, 3 Benztropine, 2 Caffeine Cannabidiol, 3 Chlorpromazine, 2 Clomipramine, 2 Clozapine, 1 Duloxetine, 1 Estradiol Fluvoxamine, 1 Haloperidol, 3 Imipramine, 2 Melatonin Mirtazapine, 3 Olanzapine, 1 Pimozide Propranolol, 1 Tacrine Thioridazine, 2	Bupropion, 1 Selegiline Sertraline, 1 Sibutramine	Amitriptyline, 1 Benztropine, 3 Cannabis, 1 Cannabidiol, 2 Citalopram, 1 Clomipramine, 1 Clozapine, 2 Diazepam Doxepin, 2 Escitalopram, 1 Estradiol Fluoxetine,3 Imipramine, 1 Nortriptyline, 2 Omeprazole Phenytoin Progesterone, 1 Sertraline, 2 Testosterone, 1 Venlafaxine, 2	Amitriptyline, 2 Benztropine, 4 Cannabidiol, 3 Cannabis, 3 Celecoxib Fluoxetine, 2 Ibuprofen, 1 Progesterone, 2 Sertraline, 3 Valproate Warfarin	Amitriptyline, 2 Amphetamine Aripiprazole, 2 Asenapine Atomoxetine Bupropion, 2 Cannabidiol, 3 Chlorpromazine, 1 Citalopram, 3 Clomipramine, 4 Clonidine Clozapine, 3 Desipramine, 4 Dextromethorphan Diphenhydramine Doxepin, 1 Escitalopram, 3 Fluoxetine, 1 Fluvoxamine, 2 Haloperidol, 1 Hydroxyzine Imipramine, 2 Mirtazapine, 2 Nortriptyline, 1 Olanzapine, 2 Paroxetine, 1 Perphenazine Propranolol, 2 Quetiapine, 2 Ranitidine Risperidone, 1 Sertraline, 2 Thioridazine, 1 Trazadone, 2 Venlafaxine, 1 Ziprasidone, 3	Alprazolam Aripiprazole, 1 Benztropine, 1 Bupropion, 2 Buspirone Cannabis, 2 Cannabidiol, 1 Cisapride Citalopram, 2 Clomipramine, 3 Clonazepam Carbamazepine Desvenlafaxine Dextromethorphan Duloxetine, 3 Escitalopram, 2 Estradiol Erythromycin Fexofenadine Guanfacine Haloperidol, 2 Loratadine Lurasidone Mirtazapine, 1 Modafinil Pimozide Progesterone, 3 Quetiapine, 1 Risperidone, 2 Sertraline, 3 Testosterone Trazadone, 1 Tiagabine Ziprasidone, 1 Zolpidem
Inhibitor	Cannabidiol Cannabis Celecoxib Cimetidine Citalopram Ciprofloxacin Clarithromycin Erythromycin Estradiol Fluvoxamine Isoniazid Ketoconazole Modafinil	Cannabidiol Cannabis	Cannabidiol Cannabis Cimetidine Contraceptives Fluconazole Fluoxetine Fluvoxamine Indomethacin Isoniazid Ketoconazole Lansoprazole Modafinil Omeprazole Oxcarbazepine Probenecid Topiramate	Cannabidiol Cannabis Cimetidine Contraceptives Fluconazole Fluoxetine Fluvoxamine Isoniazid Ketoconazole Methylphenidate Modafinil Omeprazole Paroxetine Sertraline Sulfonamides Tacrine	Asenapine Bupropion Cannabis Cannabidiol Diphenhydramine Fluoxetine Goldenseal Haloperidol Hydroxyzine Methylphenidate Paroxetine Propranolol Quinidine Ranitidine	Cannabidiol Cimetidine Ciprofloxacin Clarithromycin Cyclosporine Erythromycin Goldenseal Grapefruit juice Isoniazid Ketoconazole Prednisone Sertraline Verapamil
Inducer	Carbamazepine Cruciferous vegetables Cannabis (smoke) Char-grilling Omeprazole Phenytoin St. John’s wort Tobacco (smoke)	Carbamazepine Modafinil Phenytoin Phenobarbital Rifampin	Rifampin	Carbamazepine Phenobarbital Rifampin St. John’s wort		Carbamazepine Cruciferous vegetables Ginseng Modafinil Phenytoin Rifampin St. John’s wort

Note: Pertinent information about medications and cytochrome selectivity in Table 2 was obtained from multiple sources including literature review, [http://www.pharmgkb.org/pathways] and the Flockhart website [32]. Substrates metabolized by more than one cytochrome are numbered 1, 2, or 3 to indicate binding affinity and to delineate primary, secondary, or tertiary pathways. Inhibition in one pathway has the potential to shunt metabolism through another pathway.

## Data Availability

The data presented in this paper were obtained through public websites including literature review using PUBMED, public access websites, such as https://www.pharmgkb.org and http://drug-interactions.medicine.iu.edu [32], and standard reference books by Mrazek [38] and the PWS management book edited by Butler, Lee and Whitman [1]. The pharmacogenetic data obtained from the testing of our patients can be found in the Table A2, Table A3 and Table A4. All the tables and figures contained in this article are derived from these data.

## Appendix A

**Table A1:** Chromosome location for genes producing cytochrome P450 (CYP) enzymes, their common polymorphisms and phenotypic activity [38]; https://www.pharmgkb.org.

Cytochrome P450	Chromosome Location	Common Alleles and Phenotypic Action
*CYP2D6*	22q13.1	**1* (wildtype); **2* (normal); **2A* (increased action); **9, *10, *17, *41* (decreased action); **4, *3,* 5, *6* (inactive).
*CYP2B6*	19q13.2	**1* (wildtype); **2, *5, *17* (normal); **4, *22* (increased action); **6, *7, *9, *16, *19, *20* (decreased action); **8, *12, *13, *18,*24* (inactive).
*CYP2C19*	10q24.1–q24.3	**1* (wildtype); **11, *13, *15, *18,*28* (normal); **17* (increased action); **9, *10, *16, *19, *25, *26* (decreased action); **2-*8, *22, *24, *35-*37* (inactive).
*CYP2C9*	10q23.33	**1* (wildtype); **9* (normal); **2, *3* (decreased action); **6, *15, *25* (inactive).
*CYP3A4*	7q22.1	**1* (wildtype); **7, *9, *10* (normal); **1B, *2-*6, *8, *11-*13, *15-*18,* *22 (decreased action); **20, *26* (inactive).
*CYP1A2*	15q24.1	**1, *1A, *1B* (wildtype); **1D* (T-delT), **1F*(C/A) increased action when induced; **1C*(G/A); **1K*(C/A); **3*(T/C), *4(A/T), **7*(G/A) (decreased action).

**Table A2:** Pharmacogenetic data for cytochrome P450 enzymes in PWS cohort with Deletion: Testing laboratory, CYP genotype, and CYP phenotype.

	*CYP2D6*		*CYP2B6*		*CYP2C19*		*CYP2C9*		*CYP3A4*		*CYP1A2*	
Lab	Alleles	PT	Alleles	PT	Alleles	PT	Alleles	PT	Alleles	PT	Alleles	PT
GS	**1/*1*	EM	**1/*6*	IM	**1/*1*	EM	**1/*1*	EM	**1/*1*	EM	**1F/*1B*	UM
GS	**4/*41*	PM	**1/*6*	IM	**1/*17*	EM	**1/*1*	EM	**1/*1*	EM	**1A/*1B*	EM
GS	**2/*17* *DUP*	EM	**1/*6*	IM	**1/*17*	EM	**1/*1*	EM	**1/*1*	EM	**1A/*1B*	EM
GS	**2/*4*	IM	**1/*1*	EM	**1/*17*	EM	**1/*1*	EM	**1/*22*	IM	**1A/*1B*	EM
GS	**1/*10*	IM	**1/*6*	IM	**17/*17*	UM	**1/*1*	EM	**1/*1*	EM	**1F/*1B*	UM
GL	**2A/*5*	IM	**1/*5*	EM	**1/*1*	EM	NA	NA	**1/*22*	IM	**1A/*1A*	EM
GS	**4/*4*	PM	**1/*1*	EM	**1/*1*	EM	**1/*1*	EM	**1/*1*	EM	**1F/*1B*	UM
GS	**1/*41*	EM	**1/*1*	EM	**1/*2*	IM	**1/*1*	EM	**1/*1*	EM	**1F/*1B*	UM
GS	**1/*9*	IM	**1/*6*	IM	**1/*17*	EM	**1/*1*	EM	**1/*1*	EM	**1F/*1B*	UM
GS	**1/*9*	IM	**1/*1*	EM	**1/*17*	EM	**1/*2*	IM	**1/*1*	EM	**1A/*1B*	EM
GL	**1/*4*	IM	**1/*5*	EM	**1/*1*	EM	**1/*2*	IM	**1/*1*	EM	**1A/*1F*	HI
GL	**1/*4*	IM	**1/*6*	IM	**1/*1*	EM	**1/*1*	EM	**1/*1*	EM	**1C*1F/*1F*	HI
GS	**1/*4*	IM	**1/*1*	EM	**1/*1*	EM	**1/*2*	IM	**1/*1*	EM	**1F/*1B*	UM
GL	**1/*1*	EM	**5/*6*	IM	**1/*17*	UM	**1/*1*	EM	**1/*1*	EM	**1F/*1F*	HI

Key: Genesight (GS), Genelex (GL); PT—Phenotype; PM—poor metabolizer; IM—intermediate metabolizer; EM—extensive (normal) metabolizer; UM—ultra-rapid metabolizer; NA—result not available; HI—hyperinducer.

**Table A3:** Pharmacogenetic data for cytochrome P450 enzymes in PWS cohort with UPD: Testing laboratory, CYP genotype, and CYP phenotype.

	*CYP2D6*		*CYP2B6*		*CYP2C19*		*CYP2C9*		*CYP3A4*		*CYP1A2*	
Lab	Alleles	PT	Alleles	PT	Alleles	PT	Alleles	PT	Alleles	PT	Alleles	PT
GS	**1/*1*	EM	**1/*6*	IM	**1/*1*	EM	**1/*1*	EM	**1/*1*	EM	**1C*1D/* **1F*1B*	UM
GS	**1/*4*	IM	**1/*6*	IM	**1/*1*	EM	**1/*3*	IM	**1/*1*	EM	**1F/*1B*	UM
GS	**1/*2A*	EM	**1/*6*	IM	**1/*1*	EM	**1/*1*	EM	**1/*1*	EM	**1F/*1B*	UM
GS	**1/*41*	EM	**1/*6*	IM	**1/*17*	EM	**1/*1*	EM	**1/*1*	EM	**1A*1C/* **1B*	IM
GS	**1/*4*	EM	**1/*6*	IM	**1/*1*	EM	**1/*2*	IM	**1/*1*	EM	**1D*1E/* **1F*1B*	UM
GS	**2A/*4*	EM	**1/*1*	EM	**2/*17*	EM	**1/*1*	EM	**1/*1*	EM	**1F/*1B*	UM
GS	**1/*2A*	EM	**1/*6*	IM	**1/*17*	EM	**1/*1*	EM	**1/*1*	EM	**1F/*1B*	UM
GS	**10/*41*	PM	**1/*1*	EM	**1/*17*	EM	**1/*1*	EM	**1/*1*	EM	**1A/*1B*	EM
GS	**1/*5*	IM	**1/*1*	EM	**1/*1*	EM	**1/*2*	IM	**1/*1*	EM	**1F/*1B*	UM
GS	**1/*3*	IM	NA	NA	**1/*1*	EM	**1/*3*	IM	*NA*	NA	**1F/*1B*	UM
GS	**1/*41*	EM	**1/*6*	IM	**2/*17*	EM	**1/*1*	EM	**1/*1*	EM	**1A/*1A*	EM
GS	**4/*41*	PM	**1/*1*	EM	**1/*17*	EM	**1/*1*	EM	**1/*1*	EM	**1A/*1A*	EM
GS	**4/*41*	PM	**1/*6*	IM	**1/*1*	EM	**2/*2*	PM	**1/*1*	EM	**1F/*1B*	UM
GM	**2/*41* *Dup*	EM	**1/*6*	EM	**1/*1*	EM	**1/*1*	EM	**1/*22*	IM	**1A/*1F*	EM
GS	**1/*4*	IM	**1/*1*	EM	**1/*1*	EM	**1/*2*	IM	**1/*22*	IM	**1F/*1B*	UM
GL	**1/*2A*	EM	**2/*5*	IM	**1/*1*	EM	**1/*2*	IM	**1/*1B*	IM	**1F/*1F*	HI

Key: Genesight (GS), Genelex (GL), and Genomind (GM); PT—Phenotype; PM—poor metabolizer; IM—intermediate metabolizer; EM—extensive (normal) metabolizer; UM—ultra-rapid metabolizer; HI—hyperinducer; NA—result not available.

**Table A4:** Pharmacogenetic data for cytochrome P450 enzymes in PWS Unspecified cohort (DNA methylation positive, subtype unspecified): Testing laboratory, CYP genotype, and CYP phenotype.

	*CYP2D6*		*CYP2B6*		*CYP2C19*		*CYP2C9*		*CYP3A4*		*CYP1A2*	
Lab	Alleles	PT	Alleles	PT	Alleles	PT	Alleles	PT	Alleles	PT	Alleles	PT
GS	**2A/*4*	EM	**1/*1*	EM	**1/*1*	EM	**1/*3*	IM	**1/*1*	EM	**1A/*1B*	EM
*GS*	**1/*1*	EM	**1/*1*	EM	**1/*8*	IM	**1/*2*	IM	**1/*1*	EM	**1F/*1B*	UM
GS	**1/*1*	EM	**1/*1*	EM	**1/*1*	EM	**1/*2*	IM	**1/*1*	EM	**1A/*1B*	EM
GS	**6/*41*	PM	**1/*1*	EM	**1/*17*	EM	**1/*1*	EM	**1/*1*	EM	**1F/*1B*	UM
GS	**1/*1*	EM	**1/*1*	EM	**1/*2*	IM	**1/*1*	EM	**1/*1*	EM	**1A/*1A*	EM

Key: Genesight (GS); PT—Phenotype; PM—poor metabolizer; IM—intermediate metabolizer; EM—extensive (normal) metabolizer; UM—ultra-rapid metabolizer.

**Table A5:** Ratios of Cytochrome P450 phenotypes among PWS genetic subtypes.

PWS	*CYP2D6*	*CYP2B6*	*CYP2C19*	*CYP2C9*	*CYP3A4*	*CYP1A2*
DEL	4EM:8IM:2PM	7EM:7IM	2UM:11EM:1IM	10EM:3IM	12EM:2IM	6UM:3HI:5EM
UPD	9EM:4IM:3PM	6EM:9IM	16EM	9EM:6IM:1PM	12EM:3IM	10UM:1HI:4EM:1IM
UnSpec	4EM:1PM	5EM	3EM:2IM	2EM:3IM	5EM	2UM:3EM

Key: UM—ultra-rapid metabolizer; EM—extensive (normal) metabolizer; IM—intermediate metabolizer; PM—poor metabolizer; HI—hyperinducer; DEL—deletion; UPD—uniparental disomy; UnSpec—unspecified genetic subtype.
